# Two-Level Scheduling for Video Transmission over Downlink OFDMA Networks

**DOI:** 10.1371/journal.pone.0148625

**Published:** 2016-02-23

**Authors:** Mau-Luen Tham, Chee-Onn Chow, Yi-Han Xu, Nordin Ramli

**Affiliations:** 1 Department of Electrical Engineering, Faculty of Engineering, University of Malaya, Kuala Lumpur, Malaysia; 2 Wireless Innovation, MIMOS Berhad, Technology Park Malaysia, Kuala Lumpur, Malaysia; 3 College of Information Science and Technology, Nanjing Forestry University, Nanjing, China; Huazhong University of Science and Technology, CHINA

## Abstract

This paper presents a two-level scheduling scheme for video transmission over downlink orthogonal frequency-division multiple access (OFDMA) networks. It aims to maximize the aggregate quality of the video users subject to the playback delay and resource constraints, by exploiting the multiuser diversity and the video characteristics. The upper level schedules the transmission of video packets among multiple users based on an overall target bit-error-rate (BER), the importance level of packet and resource consumption efficiency factor. Instead, the lower level renders unequal error protection (UEP) in terms of target BER among the scheduled packets by solving a weighted sum distortion minimization problem, where each user weight reflects the total importance level of the packets that has been scheduled for that user. Frequency-selective power is then water-filled over all the assigned subcarriers in order to leverage the potential channel coding gain. Realistic simulation results demonstrate that the proposed scheme significantly outperforms the state-of-the-art scheduling scheme by up to 6.8 dB in terms of peak-signal-to-noise-ratio (PSNR). Further test evaluates the suitability of equal power allocation which is the common assumption in the literature.

## Introduction

With the rapid growth of video traffic over wireless networks, mobile operators are expected to support a large number of video subscribers simultaneously. The quality-of-service (QoS) provisioning of these services confronts two major technical hurdles. First, video communication is bandwidth intensive and delay sensitive while the inherent limited wireless resources are shared by multiple users. Second, loss of different video packets induces different amounts of distortion in the received video. When transmitting video over error-prone wireless channels, the impact of channel errors on the video can be extremely severe. To optimize the received video quality, it is essential to allocate radio resources to video packets in an efficient and robust way by taking into account the channel conditions and the video characteristics.

Resource allocation (RA) for orthogonal frequency-division multiple access (OFDMA) systems has spurred significant attention from both industry and academia communities due to high spectral efficiency and fine granularity resource control (e.g., subcarrier, power and bit). Conventional RA schemes treat video bitstreams in a content-agnostic fashion [1]. Accordingly, the measurement of QoS satisfaction level is often linked to average throughout, packet delay or/and packet loss ratio [2–5]. Such performance indicators, however, do not directly translate into actual video quality [6]. One reason is that the spatial-temporal characteristics cannot be captured in generic bitstream. Another reason lies in the fact that video bitstreams are typically equipped with source-level error robustness features. How the lost or damaged packets are concealed by a decoder dictate the final reconstructed video quality.

Several recent RA works have explicitly selected video quality as their QoS performance metric. These works can be categorized as content-aware RA methods. The core idea is to determine the importance level of each packet. In [7], a channel allocation method is devised for the quality-of-experience (QoE)-driven multimedia transmissions over the cognitive radio networks. However, power allocation is not considered. In [8], a RA scheme that maximizes the weighted sum rate of all video users is proposed. Each user weight is measured by the highest importance level of packet buffered in that user transmission queue. Resources are then distributed across multiple users as a function of user weights and channel conditions. Similar strategy is extended to the case of scalable video bitstreams in [9]. This time an additional parameter called packet deadline requirement is introduced in the weight calculation. The above two schemes, however, share the same drawback, i.e., the users with much higher weights always seize the transmission opportunity. This may lead to low resource utilization if those selected users suffer from poor channel gains on the one hand, and starvation in remaining users on the other hand. Such unfairness issue is addressed in [10] by taking into account the scheduled packet size. However, identical to [8–9], the packets from each user queue are scheduled first by neglecting the resource consumption efficiency. A step forward from allocating resources for a group of scheduled packets is the joint consideration of these two aspects. The authors in [11] demonstrate the promising video performance with such design.

Despite the significant gains accrued by the content-aware RA schemes in [8–11], their video performances are purely derived based on the assumption of error-free transmission. This, however, does not hold in practical communication systems that comprise of modulation and coding techniques. In fact, in such systems, the prime concern is to achieve the best tradeoff between the bit rate and the link reliability associated with a target bit-error-rate (BER). According to [12], the target BER criterion has a critical impact on the video performance. If the value is loose, despite the abundant availability of packets at the receiver, most of them are corrupted and thus undecodable. Instead, if the target BER is strict, packets are forwarded in a conservative way, which may render limited achievable quality though most of the packets are decodable. In [13], practical modulation and coding schemes are considered in their video transmission platform. However, similar to [12], all video packets are simply assigned with a static target BER, irrespective of the importance of that packet. Moreover, equal power allocation (EPA) is adopted to simplify the solution development.

In this paper, we propose a two-level multiuser video transmission framework which optimizes the packet scheduling and resource allocation. The ultimate goal is to maximize the average video quality of all users under the playback delay and resource constraints. This is achieved by jointly exploiting the multiuser diversity and the importance of each packet. Real encoded video bitstreams with practical modulation and coding schemes are considered. With regard to the coding aspect, a coded orthogonal frequency-division multiplexing (OFDM) system is considered. In general, any OFDM system that combines channel coding and interleaving techniques can be classified as coded OFDM systems. This technique is very effective in combating the burst errors stemming from the OFDM frequency selectivity that compromises the channel coding performance [14].

The main contributions of this paper are as follows. First, most existing RA works treat all the video packets with the same target BER. In contrast, the proposed RA allocates resources in such a way that more (less) important video packets are assigned with stricter (looser) target BERs. This can be viewed as a form of unequal error protection (UEP) which is established for its superior performance in transmission over noisy networks. Second, an overall target BER is considered to control how many average packets can be sent within one transmission interval. As observed in [12], the transmitter itself does not know the optimal value that renders the best overall video quality. In this regard, the proposed RA seeks the best quality-resource tradeoff and thus will always be a safer option irrespective of the pre-determined overall target BER.

Third, in a practical wireless video transmission system, this paper aims to cast some light on the suitability of EPA, which is the most prevalently used alleviation in the RA literature [15]. In fact, most previous works justify the use of EPA from a theoretical perspective and isolate the influence of channel coding. More specifically, validations are made by distinguishing the performance between EPA and frequency-selective power allocation (FPA) in terms of spectral efficiency, where FPA assigns power to subcarriers as a function of their channel conditions. It is however, not clear, how exactly the theoretical performance gap translates into the actual video performance gap. The findings are deemed valuable as channel coding is part and parcel of practical systems.

The rest of the paper is organized as follows. Section 2 presents the system specifications, models and assumptions. Section 3 describes the two-level scheduling design. Section 4 reports simulation details and results. Section 5 concludes the paper.

## System Model

**Fig 1:**
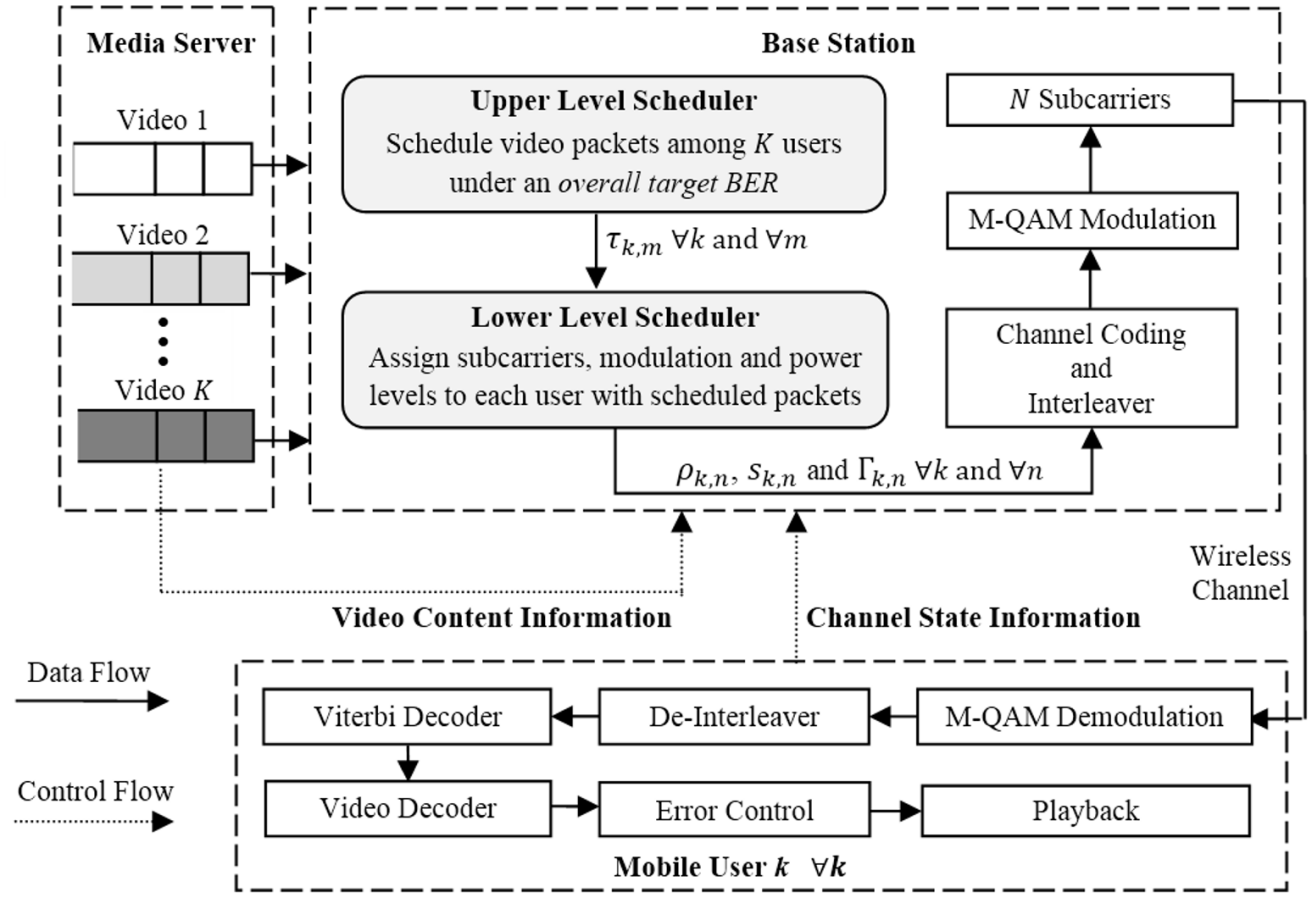
System block diagram for video over OFDMA networks.

We consider a downlink transmission scenario, where a base station (BS) transmits *K* different video streams, each to a designated user as depicted in Fig 1. The BS receives the video streams from a remote media server via a high-bandwidth and lossless connection. These streams are assumed to be pre-encoded with the H.264/AVC video coding standard [16]. Therefore, real-time transcoding techniques such as [17] are not applicable in this context. Each video frame is divided into one or more slices which can be decoded independently. We assume that one slice is directly packetized into one transport packet. Hereafter the term "slice" and "packet" will be used interchangeably. Once a user requests a video, the corresponding packets are instantly transmitted to the BS and buffered at that user queue. Each packet *m* of user *k* is stamped with the importance level *Q*_*k*,*m*_ (described later), packet size *R*_*k*,*m*_ and waiting time *T*_*k*,*m*_. These parameters are collected as video content information (VCI). Note that any packet that misses its decoding deadline *T*_*max*_ will be discarded by the BS.

All scheduled video sessions are served via a downlink OFDMA system with a total power *P*_*tot*_ and a total bandwidth *B*_*T*_. There are *N* subcarriers to be shared by all users. Each subcarrier occupies a bandwidth of *B*_*n*_ = *B*_*T*_/*N* and experiences flat fading under the assumption that *B*_*n*_ is smaller than the coherence bandwidth in a frequency-selective wireless channel. At the receiver, additive white Gaussian noise (AWGN) of power spectral density *N*_0_ with mean zero and variance *σ*^2^ = *N*_0_*B*_*n*_ is present. For user *k* on subcarrier *n*, the channel gain and the corresponding channel-to-noise ratio (CNR) are denoted by *H*_*k*,*n*_ and *γ*_*k*,*n*_ = |*H*_*k*,*n*_|^2^/*σ*^2^, respectively.

We assume that the BS has perfect knowledge of channel state information (CSI) and the channel conditions remain static over one time transmission interval (TTI). The TTI corresponds to the time length of a single media access control (MAC) subframe with *L* OFDMA symbols for payload transmission. Note that although “frame” is a more accurate way to refer to the physical layer service data unit (SDU), we use “subframe” to avoid confusion between MAC and video frames. Then, the achievable transmission bits at subframe *t r*_*k*,*n*_[*t*] is expressed as

$$\;r_{k,n}\left[t\right]=\;L{\text{log}}_{2}(1+\;\alpha_{k,n}\left[t\right]\gamma_{k,n}\left[t\right]s_{k,n}\left[t\right])\;\forall k,\forall n.$$ (1)

where *α*_*k*,*n*_ and *s*_*k*,*n*_ denote realistic coding gap and transmission power allocated on subcarrier *n* of user *k*, respectively. If square M-QAM modulation with Gray bit mapping is adopted, *α*_*k*,*n*_ is provided by

$$\alpha_{k,n}\left[t\right]=-1.5/\text{ln}\left(5BER_{k,n}\left[t\right]\right),\;\alpha_{k,n}\geq 0\;\forall k,\forall n.$$ (2)

[18]. Note that *r*_*k*,*n*_[*t*] should be restricted to integer form due to discrete modulation levels Γ_*k*,*n*_ which is given by

$$\Gamma_{k,n}\left[t\right]=\;1+\alpha_{k,n}\left[t\right]\gamma_{k,n}\left[t\right]s_{k,n}\left[t\right]\;\forall k,\forall n.$$ (3)

However, we assume a non-integer number of bits for tractability first. This constraint will be explicitly considered in Section 3–2. With *r*_*k*,*n*_[*t*], the number of video bits that can be loaded onto subcarrier *n* is expressed by

$${\bar{r}}_{k,n}\left[t\right]=c\times r_{k,n}\left[t\right]\;\forall k,\forall n.$$ (4)

where *c* is the coding rate.

Denote *ρ*_*k*,*n*_ as the indicator for subcarrier allocation, where *ρ*_*k*,*n*_ = 1 implies that subcarrier n is assigned to user *k*; otherwise, *ρ*_*k*,*n*_ equals to zero. As each subcarrier cannot be shared by multiple users, the binary variable ρ_k,n_ should satisfy

$$\rho_{k,n}\in \left\{0,1\right\},\;\sum_{k=1}^{K}\rho_{k,n}=1\;\forall n.$$ (5)

Let Ω_*k*_ be the set of subcarriers assigned to user *k*. Then, only subcarrier *n* with ρ_k,n_ = 1 is included in Ω_*k*_. To ease the notational burden, let overall CNR as a matrix **γ** with [**γ**]_*k*,*n*_ *=* γ_*k*,*n*_, rate allocation as a matrix **r** with [**r**]_*k*,*n*_ *= r*_*k*,*n*_, subcarrier assignment as a matrix ρ with [**ρ**]_*k*,*n*_ = *ρ*_*k*,*n*_, power allocation as a matrix **s** with [**s**]_*k*,*n*_ = *s*_*k*,*n*_, and coding gap as a matrix ***α*** with [***α***]_*k*,*n*_ = ***α***_*k*,*n*_.

Based on the video content information and the channel state information, the proposed two-level scheduler (shaded area) makes decision once every subframe. The upper level schedules the transmission of video packets among multiple users based on an overall target bit-error-rate (BER). Instead, the lower level assigns subcarriers to the scheduled users, as well as adjusts modulation and power levels of each subcarrier. The ultimate goal is to maximize the average overall quality of all users while adhering to resource and playback constraints. Once the optimization decision is made, each scheduled user flow undergoes bit-interleaved coded modulation (BICM) in isolation due to its strong forward error protection in frequency-selective fading environments [14].

Let us now scrutinize the BICM operation in the context of video delivery. First, the selected packets from video *k* are aggregated into a bitstream which will be convolutionally encoded into coded bits and then interleaved across several subcarriers that has been assigned to that corresponding user. The number of interleaved bits that can be loaded onto each subcarrier depends on the calculated modulation constellation size. Second, at the receiver, hard-decision demodulation is performed on the received signal of each subcarrier, followed by de-interleaving in order to assemble the coded bits. Third, the concatenate video bitstream is obtained by passing the coded bits to the Viterbi decoder. Finally, the bitstream is unmerged into video packets where only error-free packets are passed onto the video decoder and corrupted ones are considered lost.

Error concealment invokes when there is any lost packet in order to mask the visual artifacts. We choose the copy-from-previous (CP) error concealment method due to its simplicity and efficiency [19]. In particular, it copies the pixel values of the entire slice at the co-located slice from the previously decoded frame. We then define *Q*_*k*,*m*_ as the quality increment of decoded video *k* caused by the transmission of packet *m*. Mathematically, it can be expressed as

$$Q_{k,m}=D_{k,m}^{L}-D_{k,m}^{T}$$ (6)

where $D_{k,m}^{L}≜D_{k}\left(\tau_{k,m}=0{|\Pi }_{k}\left(m-1\right)\right)$ and $D_{k,m}^{T}≜D_{k}\left(\tau_{k,m}=1{|\Pi }_{k}\left(m-1\right)\right)$ are the distortion caused by the lost and the transmission of packet *m* after the transmission of Π_*k*_(*m*−1)≜{*π*_k,1_,*π*_k,2_,…,*π*_k,m−1_}, respectively. *τ*_k,m_ is the indicator for packet scheduling, where *τ*_k,m_ = 1 implies that packet *m* of user *k* is scheduled at the current subframe. Otherwise, *τ*_k,m_ = 0. Π_*k*_(*m*−1) denotes the reordered set of packets in user queue *k* such that *π*_k,1_ is the first packet to be transmitted from that queue.

A brute-force approach that obtains *Q*_*k*,*m*_ in Eq (6) is to calculate the mean square error (MSE) of pixel values between original and concealed frames. This, however, poses a prohibitive computational burden at the BS due to the nontrivial decoding process. For this reason, we resort to the transmission distortion model as proposed in [20], where $D_{k,m}^{L}$ and $D_{k,m}^{T}$ are predicted by using a different function of expected distortion accumulated up to the previous frame *f*−1 (denoted as $E\left[D_{k,m}^{f-1}\right]$), macroblock (MB) partition modes and motion vector. Obviously, all these parameters can be readily obtained during the video encoding process except that the expected distortion must be computed on the fly in order to capture the effect of error propagation.

Note that other transmission distortion models associated with different error concealment methods can potentially be incorporated here, but they are not investigated due to space limitations. Also, we do not consider retransmission of erroneous packets as it may not be feasible for real-time video applications.

## Two-Level Scheduling for Video Transmission

In this section, we present a two-level scheduling scheme for video transmission. First, we discuss the packet scheduling scheme. Second, we formulate an optimization problem for assigning subcarriers to the scheduled packets of different users and then assign modulation levels as well as power on those subcarriers. Finally, we elucidate how to update *Q*_*k*,*m*_.

### Packet Scheduling

The goal of packet scheduling is to sort the video packet transmission order as a function of the importance level *Q*_*k*,*m*_ and *J*_*k*,*m*_, at the beginning of each subframe. *J*_*k*,*m*_ denotes the resource consumption level of packet *m*. For each user queue, there are *M*_*k*_ available packets of the same frame at subframe *t*. In general, the number of subframes available per video frame can be calculated by ⌊*T*_*max*_/TTI⌋. Hereafter we omit the time index “*t*” for simplicity as it remains invariant throughout this paper.

Based on the idea in [11], *J*_*k*,*m*_ is defined as the least number of free subcarriers needed to send packet *m* with the premise of EPA. Then, the packet with the best ratio of *Q*_*k*,*m*_/*J*_*k*,*m*_ is served in one selection loop until there are no more subcarriers. The procedures are summarized as follows:

**Algorithm 1: Packet Scheduling.**

1: Initialization: Set **N** = {1, 2, *itN*} and **M**_**k**_ = {1, 2, it*M*_*k*_}for ∀*k*; obtain *Q*_*k*,*m*_ for ∀*m* ∈ **M**_**k**_ and ∀*k*; set *BER*_*k*,*n*_ = *overall target BER* for ∀*k* and ∀*n*; set *s*_*k*,*n*_ = *P*_*tot*_/*N* for ∀*k* and ∀*n*.

2: **repeat**

3:  **for** *k* = 1 to *K*

4: Sort *γ*_*k*,*n*_ of set **N** in a descending order. Obtain corresponding mapping indexes *I*_*k*_(*n*) ∈ {1,2,*α N*} for ∀*n*

5: Using Eq (4), construct set $A_{k}=\left\{{\bar{r}}_{k,1},{\bar{r}}_{k,2},\ldots ,{\bar{r}}_{k,\left|N\right|}\right\}$ corresponding to the reordered subcarrier indexes. Furthermore, construct set $\bar{A_{k}}=\left\{\bar{r_{k,1}},\bar{r_{k,2}},\ldots ,\bar{r_{k,\left|N\right|}}\right\}$ by computing the cumulative sum of the elements along ***A***_***k***_.

6:   **for** *m* = 1 to *M*_*k*_

7:   Find n* such that $\bar{r_{k,n-1}}≺R_{k,m}≼\bar{r_{k,n}}$ and set *J*_*k*,*m*_ = n*

8:   **end for**

9:  **end for**

10: Find k*,m* = arg max_*k*,*m*_(*Q*_*k*,*m*_/*J*_*k*,*m*_) and set *τ*_k*,m*_ = 1

11: Update **M**_k*_ = **M**_k*_−m*; **N** = **N**−{*I*_k*_(1), *I*_k*_(2), …, *I*_k*_(*J*_k*,m*_)}

12: **until** no more packet to be streamed or the condition of line 07 is not satisfied for ∀*k*∀*m*

Switching the *overall target BER* may change not only the number of scheduled packets, but also the packet order to be transmitted. This is because *J*_*k*,*m*_ is associated with the transmission rate that varies accordingly with the *overall target BER*. Hence, [11] that does not consider *overall target BER* can be treated as a special case of *Algorithm 1*.

From the BS perspective, it would seem logical that the packets are forwarded as fast as possible (by setting *overall target BER* to a loose value) in order to avoid deadline violations. However, the pitfall is that many received packets are possibly corrupted and thus undecodable. On the other hand, if packets are forwarded in a conservative way, it may render limited achievable video quality though most of the packets are useful. This is in line with the observation in [12], that *overall target BER* criterion has a critical impact on the video performance and its optimal value cannot be known a priori. Under such circumstances, resource allocation should be adaptive to the scheduled packet priority in an UEP way so that the average overall video distortion can be minimized.

### Resource Allocation

To bridge the gap, we propose an UEP-oriented subcarrier assignment strategy that assigns stricter target BERs to more important packets and *vice versa*. To this end, an optimization problem that minimizes a weighted sum of user coding gaps subject to the scheduled packet length constraints is formulated. Assuming EPA, the problem can be expressed as

$$\underset{\left\{\rho ,\alpha \right\}}{\text{min}}\sum_{k=1}^{K}\sum_{n=1}^{N}\omega_{k}\rho_{k,n}\alpha_{k,n}$$

subject to Eqs (2) and (5),

$$\sum_{n=1}^{N}\rho_{k,n}r_{k,n}\geq \frac{1}{c}\sum_{m=1}^{M_{k}}\tau_{k,m}R_{k,m}\forall k$$ (7)

where *ω*_*k*_ denotes the weight factor for user *k*. The last constraint implies that the resources allocated to user *k* should be adequate enough to transmit the video packets scheduled from *Algorithm 1*. We then project the importance level of video packets into the weight, which is given by

$$\omega_{k}=\sum_{m=1}^{M_{k}}\tau_{k,m}Q_{k,m}\forall k$$ (8)

It is apparent that a group of more important packets from user *k* will lead to a larger *ω*_*k*_. Thus, user *k* has higher priority to occupy a subcarrier such that their corresponding *α*_*k*,*n*_ is reduced. However, user *k* does not essentially dominate all the best subcarriers. This is possible when another user who happens to have only a few good subcarriers that overlap with those of user *k*. Furthermore, the link reliability of a packet is tightly connected with the modulation order which is adopted to carry that packet. If user *k* already obtains sufficient subcarriers (with low modulation orders) to reliably transmit all data, remaining subcarriers can be granted to other users.

All these essences are captured in problem Eq (7) and solving it actually translates into a reduction in the average distortion of all scheduled packets. As *α*_*k*,*n*_ is an increasing function of *BER*_*k*,*n*_, solution to Eq (7) yields to a situation where more important packets are possibly error-free due to stricter BERs on the one hand, and less critical packets are exposed to higher BERs on the other hand. Fortunately, the amounts of quality degradation for those low-priority packets are anticipatively minimal as they are easily concealable. The relationship between *α*_*k*,*n*_ and *BER*_*k*,*n*_ is proved in Appendix.

Note that Eq (7) has to be solved in every subframe and so does the update of *ω*_*k*_ associated with *Q*_*k*,*m*_. Since *Q*_*k*,*m*_ depends on the past scheduling decisions, solution to Eq (7) offers means of effecting long-term fairness among different users in the following sense. If scheduled packets of user *k* are assigned loose target BER in the current subframe, the current video frame may suffer more distortion that would propagate to subsequent frames due to the use of predictive coding. In response to the loss, *Q*_*k*,*m*_ of the new frame would essentially grow larger. This enforces the BS to assign stricter BER to user *k*’s packets in the future subframes.

To solve Eq (7), let us denote *μ*_*k*_ as the Lagrange multipliers for the rate constraints and let [**μ**]_*k*_ = *μ*_*k*_ be the multiplier vector. Then, the problem Eq (7) is equivalent to finding the minimum of the following Lagrange function

$$ℒ\left(\rho ,\;\alpha ,\;\mu \right)=\sum_{k=1}^{K}\sum_{m=1}^{N}\omega_{k}\rho_{k,n}\alpha_{k,n}-\sum_{k=1}^{K}\mu_{k}\left(\sum_{n=1}^{N}\rho_{k,n}r_{k,n}-\left(\frac{1}{c}\sum_{\text{m}=1}^{M_{k}}\tau_{k,m}R_{k,m}\right)\right)$$ (9)

The above problem can be solved efficiently with the Lagrange dual decomposition method as long as the number of subcarriers becomes sufficiently large, i.e., *N* ≥ 8, which renders the duality gap negligible [21–22]. For this, we define the dual function *g*(**μ**) as

$$g(\mu )=\underset{{}_{\{\rho ,\;\alpha \}\in \Lambda }}{\text{min}}ℒ(\rho ,\;\alpha ,\;\mu )$$ (10)

where the domain Λ denotes the set of **ρ** satisfying constraint Eq (5) and **α** satisfying constraint Eq (2). Then, the dual problem can be expressed as

$${\underset{}{\text{max}}}_{\mu \leq 0}\;g\left(\mu \right)≜\sum_{n=1}^{N}g_{n}^{\text{'}}\left(\mu \right)+\sum_{k=1}^{K}\mu_{k}\left(\frac{1}{c}\sum_{\text{m}=1}^{M_{k}}\tau_{k,m}R_{k,m}\right)$$ (11)

where *g*(**μ**) is decomposed into *N* independent problems $g_{n}^{\prime }\left(\mu \right)$ which is defined by

$$g_{n}^{\prime }\left(\mu \right)=\;\underset{\left\{\rho ,\;\alpha \right\}\in \Lambda }{\text{min}}\left\{\sum_{k=1}^{K}\rho_{k,n}\left(\omega_{k}\alpha_{k,n}-\mu_{k}r_{k,n}\right)\right\}\forall n.$$ (12)

To solve Eq (12) individually for *n* = 1, …, *N* under the integer constraint of *ρ*_*k*,*n*_, the subcarrier *n* should be assigned to queue *k* with the minimum value of *F*_*k*,*n*_(*α*_*k*,*n*_)≜(ω_*k*_*α*_*k*,*n*_−*μ*_*k*_*r*_*k*,*n*_). For this, we take the derivative of $\sum_{k=1}^{K}F_{k,n}\left(\alpha_{k,n}\right)$ with respect to *α*_*k*,*n*_ which results in

$$\alpha_{k,n}^{\prime }={\left[\frac{L\mu_{k}}{\text{ln}2}-\frac{1}{\gamma_{k,n}s_{k,n}}\right]}^{+}\forall k,\;\forall n,$$ (13)

where [*x*]^+^ is defined as max(*x*, 0). Then, we derive the subcarrier assignment $\rho_{k,n}^{\prime }$ as

$$\rho_{k,n}^{\prime }=\{\begin{array}{cc} 1, & k=\underset{k}{\text{argmin}}F_{k,n}\left(\alpha_{k,n}^{\prime }\right)\\ 0, & \text{otherwise}. \end{array}\;\forall n.$$ (14)

We now replace Eqs (13) and (14) into Eq (12) and rewrite Eq (12) as

$$g_{n}^{\prime }\left(\mu \right)=\;\sum_{k=1}^{K}\rho_{k,n}^{\prime }\left(\omega_{k}\alpha_{k,n}^{\prime }-\mu_{k}r_{k,n}^{\prime }\right)\;\forall n$$ (15)

where $r_{k,n}^{\prime }$ is obtained using Eq (2) with $\alpha_{k,n}=\rho_{k,n}^{\prime }\alpha_{k,n}^{\prime }$. After solving Eq (15) individually for *n* = 1, …, *N*, *g*(**μ**) is computed from Eq (11). To find the optimal μ* that maximizes Eq (1) regardless of the dual function differentiability, the update of **μ** can be done with the subgradient method in which the subgradient for queue *k* is defined as

$$\Delta G_{k}=\frac{1}{c}\sum_{m=1}^{M_{k}}\tau_{k,m}R_{k,m}-\sum_{n=1}^{N}r_{k,n}^{\prime }\;\forall k$$ (16)

where $\rho_{k,n}^{\prime }$, $\alpha_{k,n}^{\prime }$ and $r_{k,n}^{\prime }$ are the optimizing variables in the definition of *g*(**μ**) = *ℒ*(**ρ, α, μ**). Then, **μ** is updated by

$$\mu_{k}^{i+1}={\left[\mu_{k}^{i}+\delta^{i}\Delta G_{k}^{i}\right]}^{+}\forall k$$ (17)

where *i* is the iteration index, *δ*^*i*^ is a positive step size which follows diminishing step size policy [23], and $\Delta G_{k}^{i}$ is the subgradient of *g*(**μ**) for queue *k* at current iteration *i*. The same iterative procedure is repeated until the subgradients of all queues converge to zero. Finally, μ = μ*allows us to attain near optimal solution ρ* ≈ ρ′ and α* ≈ α′ because the strong duality holds. The complete steps are given in *Algorithm 2*. Denote *Z*_1_ as the number of iterations needed to update $\mu_{k}^{i}$ until $\left|\Delta G_{k}^{i}\right|<\epsilon$. Then, the complexity is $O\left(Z_{1}KN\right)$.

**Algorithm 2: Subcarrier Assignment.**

1: Initialization: Set *i* = 1, **μ**^*i*^>0, and choose 0 < *ϵ* ≪ 1 as the stopping criterion.

2: **repeat**

3: Set *ρ*_*k*,*n*_ = 0∀*k*,∀*n*.

4: **for** *n* = 1 to *N*

5:  **for** *k* = 1 to *K*

6:  Compute $\alpha_{k,n}^{\prime }$ using Eq (13).

7:  **end for**

8: Set $\rho_{k,n}^{\prime }$ using Eq (14).

9: **end for**

10:*i* = *i*+1

11: Update $\mu_{k}^{i}$ according to Eq (17) ∀*k*

12: **until $\left|\Delta G_{k}^{i}\right|<\epsilon \forall k$**

So far we assume continuous version of modulation levels. In practice, the level can be only selected from the following set $\Gamma_{k,n}\in \left\{0,\ldots ,2^{b},\ldots ,2^{b_{max}}\right\}$. *b* represents an even number starting from *b* = 2 and stands for the number of bits. *b*_*max*_ is the maximum number of bits and its corresponding $2^{b_{max}}$ is the highest modulation level. Note that Γ_*k*,*n*_ *=* 0 indicates no transmission.

Based on the objective function in Eq (7) with the assumption of EPA, an obvious way is to assign next modulation level to the subcarrier which requires the lowest additional coding loss factor α_*k*,*n*_. However, it may not yield good video quality as the Viterbi decoder views the whole subchannel (those subcarriers that have been assigned to user *k*) as a channel with fluctuating BERs associated with *SNR*_*k*,*n*_ = *s*_*k*,*n*_*γ*_*k*,*n*_. It is well known that channel coding performs poorly in such environments and frequency-selective power allocation (FPA) can enhance the performance [14].

By using the greedy bit loading algorithm in [24], we assign next modulation level to the subcarrier with the smallest power increment. For each scheduled user, this process repeats for a total iteration of $\left(\frac{1}{c}\sum_{m=1}^{M_{k}}\tau_{k,m}R_{k,m}\right)/2$, where one iteration requires |Ω_*k*_| comparisons to search for the most power-efficient subcarrier. Once this technique is performed for every scheduled user, all subcarriers’ power is normalized such that *P*_*tot*_ is satisfied. The overall complexity is given as $O\left(\left(\sum_{k=1}^{K}\left(\frac{1}{c}\sum_{m=1}^{M_{k}}\tau_{k,m}R_{k,m}\right)\times \left|\Omega_{k}\right|\right)/2\right)$.

The power allocation strategy deserves further attention. So far in the RA literature, EPA is the most commonly used assumption to alleviate the computational complexity. While such simplification enjoys the (near) optimal performance in efficiency-oriented resource allocation policies, this is not the case for fairness-oriented schemes [15]. The reason is that when fairness is emphasized, some bad channel users have to be scheduled that culminates in high channel gain variations among the users. In this case, FPA is necessary as the achieved performance gain can be significant [25]. However, these works justify their own arguments from the theoretical perspective and isolate the influence of channel coding. In contrast, we investigate the necessity of FPA from the video performance perspective.

### *Q*_*k*,*m*_ Update

Recall that *Q*_*k*,*m*_ estimation requires the expected distortion accumulated up to the previous frame *f*−1, which is given by

$$E\left[D_{k,m}^{f-1}\right]=\left(1-P_{k,m}^{f-1}\right)D_{k,m}^{T\left(f-1\right)}+P_{k,m}^{f-1}D_{k,m}^{L\left(f-1\right)}$$ (18)

where $P_{k,m}^{f-1}≜1-{\left(1-BER_{k,m}^{f-1}\right)}^{R_{k,m}^{f-1}}$ is the loss probability of packet *m* of frame *f*−1. If the packet is dropped by the BS, $P_{k,m}^{f-1}$ is set to one as it is assumed lost. Otherwise, $P_{k,m}^{f-1}$ associated with $BER_{k,m}^{f-1}$ can be computed using Eq (2) since we already have the past scheduling decisions (i.e., Γ_*k*,*n*_ and *s*_*k*,*n*_) by executing the proposed two-level scheduling scheme. From Eq (18), it is clear that $E\left[D_{k,m}^{f-1}\right]$ is larger if that packet is assigned with larger BER as compared to smaller BER. Thus, *Q*_*k*,*m*_ of the new frame would react to become larger in order to reflect its urgency in terms of BER protection level.

## Results and Discussion

In the simulations, six video sequences with 100 frames: *foreman*, *carphone*, *coastguard*, *silent*, *mobile* and *news* in QCIF (176 × 144) format are used. The video sequences are encoded in H.264 (JVT reference software, JM 18.4 [26]), with the baseline profile, a frame rate of 25 fps and an encoding rate of 350 kbps. All frames, except for the first frame, are encoded as P frames. Each slice consists of a row of MBs. To enhance error robustness, random I MBs are inserted into each frame, and constrained intra-prediction is activated. The playback delay budget *T*_*max*_ of each packet is 40 ms.

For the OFDMA, the aggregate bandwidth *B*_*T*_ is 1 MHz, the available transmit power *P*_*tot*_ is 1 W and the number of subcarriers *N* is 256. The wireless channel is modeled as a frequency-selective fading channel that consists of six independent Rayleigh multipaths. Each multipath component is modeled by Clarke's flat fading model [27]. We assume the power delay profile exponentially decays with *e*^-2v^, where *v* is the multipath index. Then, the relative power of the six multipath components are [0,−8.69,−17.37,−26.06,−34.74,−43.43] dB. The length of TTI is 5 ms which corresponds to 19 OFDM symbols. We assume that *L* = 15 out of 19 symbols are adopted for packet transmission while the rest is supposed to account for the signalling overhead. The maximum Doppler shift is 30 Hz and the power spectral density of the white Gaussian noise is -80 dBW/Hz. The channel coding parameters are the same as in [12], in which the convolutional encoder employs *c* = 1/2 with constraint length 5 and code generator polynomial of [23, 35] in octal. We assume four different MQAM constellations **Γ** = {0, 4, 16, 64, 256} corresponding to 0, 2, 4, 6, and 8 bits per symbol. For each subcarrier *n* assigned to user *k*, *SNR*_*k*,*n*_ is computed and channel errors are inserted in the modulated bitstream according to *SNR*_*k*,*n*_. The average SNR of all users is 15 dB.

To assess the decoded video quality, we use PSNR ≜ 10log_10_(255^2^/MSE). For each video, the PSNR as reported below is averaged over 100 frames and over five sets of decoded video corresponding to five random error patterns for each time slot. All results are included in dataset (S1 Dataset). Four schemes are compared as follows.

1. UEP-CA: This is the proposed two-level scheduling scheme. Recall that UEP is enabled within all scheduled packets via resource allocation.
2. UEP-CA-EPA: This is the same as UEP-CA except that EPA is executed.
3. CA [11]: This scheme is selected as the representative content-aware (CA) RA since it outperforms the other CA schemes (e.g. [8–9]), as reported in [11] itself. The same packet scheduling as in UEP-CA runs, but it directly assigns subcarriers to different users with EPA. Thus, all scheduled packets share the same BER protection level. Then, EPA is employed.
4. MAX [28]: The classical sum rate maximization serves as a benchmark. It assigns subcarriers to the user with the best channel gain on it with EPA. In other words, it is a content-blind RA.

The complexity of each scheme is summarized in Table 1.

**Table 1:** Complexity analysis for different algorithms.

Scheme	Computational Complexity
UEP-CA/ UEP-CA-EPA	C_1_+C_2_+C_3_+C_4_
CA [11]	C_1_+C_2_+C_4_
MAX [28]	C_5_+C_4_

${\text{C}}_{\text{1}}=O\left(KM\right)$ corresponding to the complexity for computing *Q*_*k*,*m*_ prior to scheduling.

${\text{C}}_{\text{2}}=O\left(N\left(K\;{\text{log}}_{2}N+KM_{k}\right)\right)$ corresponding to *Algorithm 1*.

${\text{C}}_{\text{3}}=O\left(Z_{1}KN\right)$ corresponding to *Algorithm 2*.

${\text{C}}_{\text{4}}=O\left(\left(\sum_{k=1}^{K}\left(\frac{1}{c}\sum_{m=1}^{M_{k}}\tau_{k,m}R_{k,m}\right)\times \left|\Omega_{k}\right|\right)/2\right)$ corresponding to discrete modulation.

${\text{C}}_{\text{5}}=O\left(KN\right)$ corresponding to max-gain subcarrier assignment.

**Fig 2:**
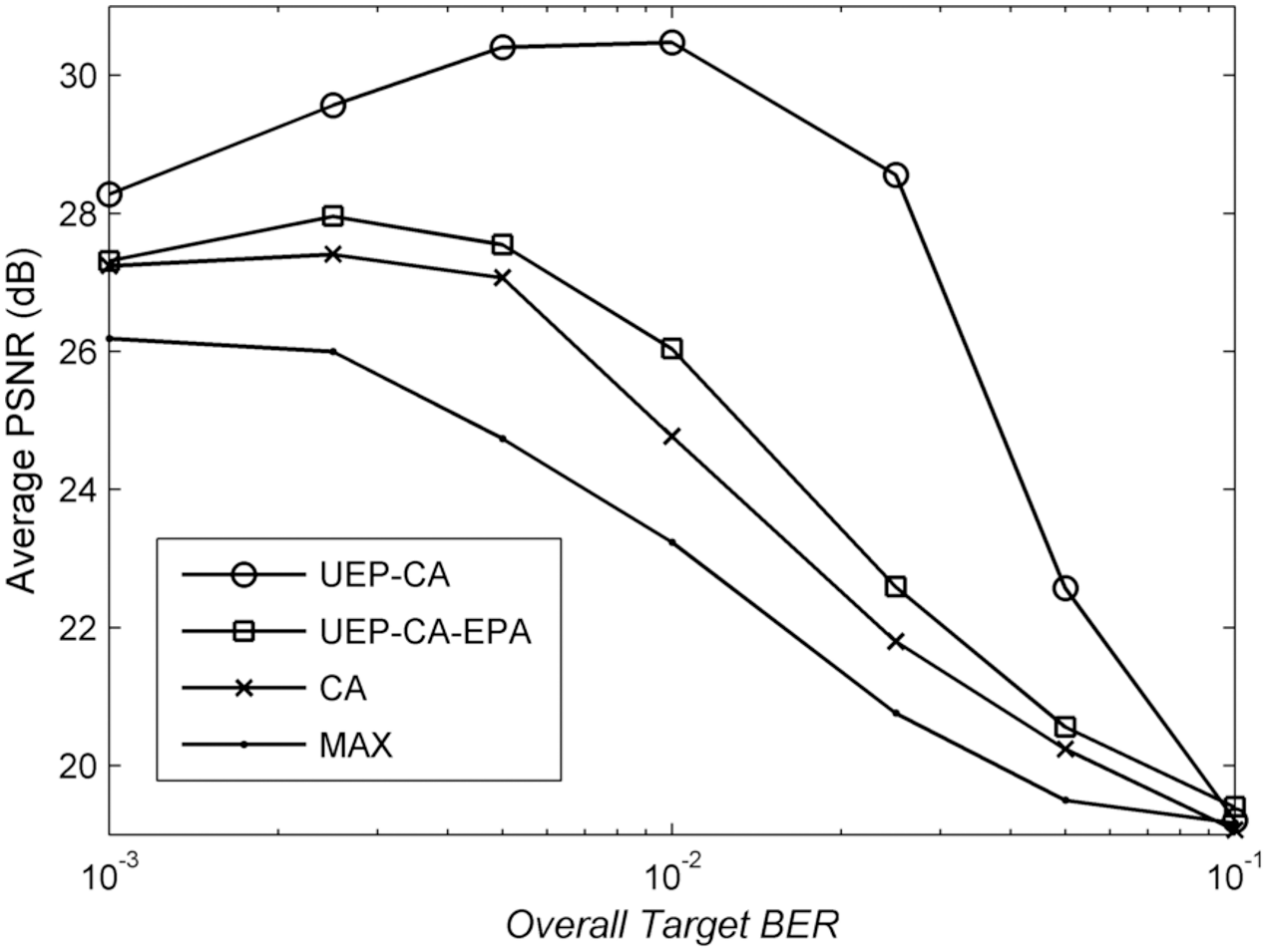
Average PSNR over all users versus *overall target BER* for different schemes.

Fig 2 shows the average PSNR over all six video sequences for different *overall target BER*s and methods. It can be seen that the PSNR curves for all schemes possess different optimal *overall target BERs*. Nevertheless, all the curves tend to drop when *target BER* loosens. Reaching at 10^−1^, all methods have similar poor performance of 19 dB. The reason is that higher modulation levels are needed to accommodate a burst of video packets under this setting. Therefore, most of them are corrupted and thus undecodable. It is true that the two proposed schemes especially UEP-CA significantly outperforms both CA and MAX for the entire BER range, reaching up to 6.8 dB and 7.8 dB, respectively. In other words, the proposed scheme will always be a safer option irrespective of the pre-determined *overall target BER* due to its robustness against channel errors.

In addition, the advantage of FPA over EPA can be observed from the PSNR gap between UEP-CA and UEP-CA-EPA, where the highest gap is 5.9 dB. Therefore, EPA could be an inappropriate choice and FPA is necessary to fully harness the channel coding performance. Interestingly enough, under the same EPA approach, UEP-CA-EPA is still better than CA for the entire BER range, with the gain as high as 1.3 dB. This indicates that the performance improvement does not solely derive from the FPA. As *Algorithm 2* assigns more subcarriers associated with lower modulation levels to higher-priority packets, it guarantees a certain level of link reliability. Besides, as expected, MAX yields the poorest performance as it does not consider any video-related information.

**Fig 3:**
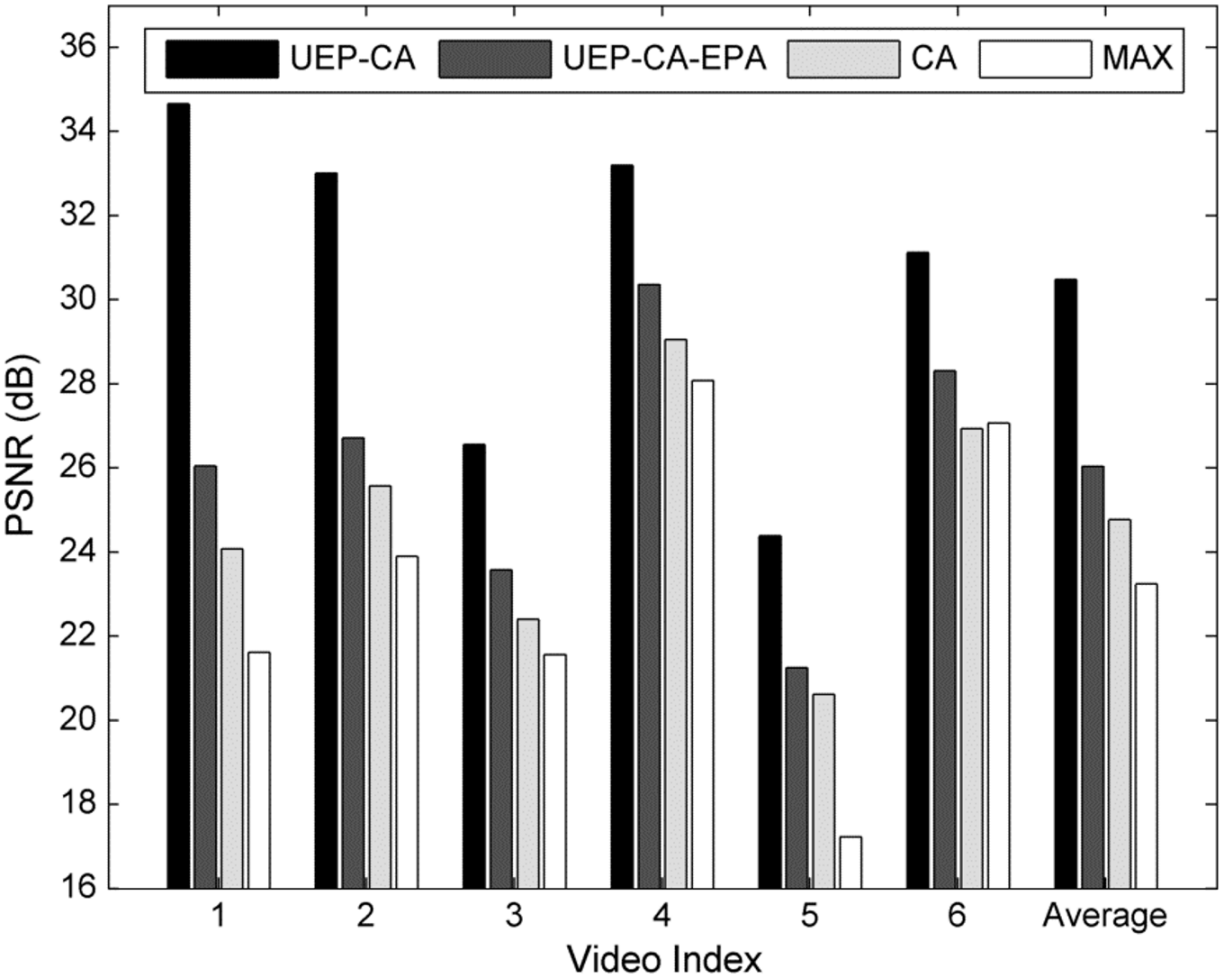
Average PSNR per video for different schemes at *overall target BER* = 10^−2^. Video index represents 1: *foreman*, 2: *carphone*, 3: *coastguard*, 4: *silent*, 5: *mobile* and 6: *news*.

Fig 3 depicts the average PSNR for each video corresponding to *overall target BER* = 10^−2^. It is obvious that UEP-CA-like schemes are superior to their counterparts over all video sequences. A larger performance improvement can be found not only in high-motion (complex) sequences such as *foreman* and *carphone* but also in slow-motion (simple) videos such as *news*. The packets of complex video are generally more important that those of simple video. Thus, many complex packets are likely to be transmitted where the benefit of protecting these packets via stricter target BER is more visible. On the other hand, when the simple packets are scheduled, they are deemed extremely important since many inter-coded packets rely on them. The effect of error propagation can last for a long period if these packets are distorted. UEP-CA-like schemes recognize this fact by assigning stricter target BERs to them.

**Fig 4:**
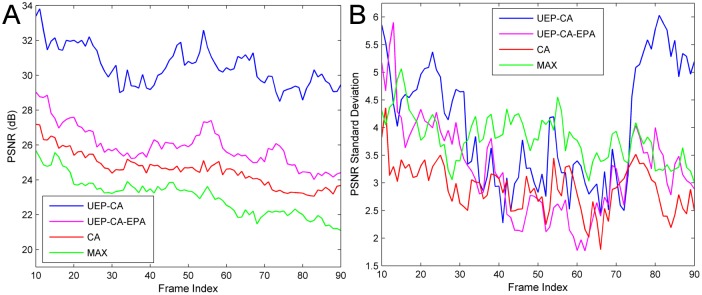
Frame-by-frame quality over all users for different schemes at *overall target BER* = 10^−2^. (a) Average PSNR per frame. (b) PSNR variance across all users.

To gain insight of the performances from another perspective, Fig 4 depicts the average PSNR and the standard deviation (STD) of the PSNR per frame over all clients for all comparison schemes corresponding to *overall target BER* = 10^−2^. For easeof exposition, we only provide the outcome from the 10^th^ to 90^th^ frame.

From Fig 4(a), it is observed that UEP-CA-like schemes consistently perform better than their alternatives over all frames. From Fig 4(b), it can be seen that CA tends to render relatively identical video quality per frame across all the clients. Such performance trend is actually not a good sign as it is attributed to its relatively low average PSNR. While the STD of UEP-CA is only -0.8~3.5 dB as compared to CA, UEP-CA shows a remarkable PSNR gain of 4.3~7.5 dB over CA. In other words, UEP-CA at its peak accrues a total quality improvement of (7.5×4 = 45 dB) over CA. Also, the video itself has its own initial average PSNR due to the same encoding rate. Therefore, such slight degradation in terms of video fairness for the UEP-CA case is reasonable. In fact, the results in Fig 3 suggest that UEP-CA-like schemes ultimately lead to the best average quality of 100 frames for all videos.

Additionally, a very meaningful phenomenon is found at the STD curves around the 80^th^ frame for the UEP-CA. There is a steep rise and drop centered at that point. The rising STD prior to the pivotal point (75^th^~79^th^) indicates that certain users were either suffering resource starvation or lacking of BER protection at that time. This issue would be compensated by *Algorithm 2* where stricter BERs would be assigned to 81^st^~85^th^ frames of those users.

## Conclusions

We have proposed a two-level scheduling framework for transmitting H.264 videos over multiuser coded OFDM systems, by taking into account the channel conditions, the video information and multiple target BERs. The main contribution lies in how UEP of video packets is rendered via subcarrier assignment and power allocation strategies. A novel weighted sum distortion minimization problem was formulated and frequency-selective power allocation was employed. Bit-level simulation results demonstrate that the proposed schemes remarkably outperform the advanced schemes, with the PSNR gain as high as 6.8. Furthermore, via comparison with equal power allocation, we recommend that frequency-selective power allocation is necessary to fully exploit the potential channel coding gain.

## Appendix

### Relationships between *α*_*k*,*n*_ and *BER*_*k*,*n*_

Applying the natural logarithm on both sides of Eq (2), we obtain

$$\text{ln}\alpha_{k,n}=\;\text{ln}\left(\frac{1.5}{-\text{ln}\left(5BER_{k,n}\right)}\right)$$ (19)

$$=\text{ln}1.5-\text{ln}\left(-\text{ln}\left(5BER_{k,n}\right)\right)$$ (20)

We differentiate Eq (20) with respect to *BER*_*k*,*n*_ to get

$$\left(\frac{1}{\alpha_{k,n}}\right)\left(\frac{\partial \alpha_{k,n}}{\partial BER_{k,n}}\right)=-\left(\frac{1}{-\text{ln}\left(5BER_{k,n}\right)}\right)\left(\frac{1}{BER_{k,n}}\right)$$ (21)

After some algebraic operations, Eq (21) can be rewritten as

$$\left(\frac{\partial \alpha_{k,n}}{\partial BER_{k,n}}\right)=\;\left(\frac{1.5}{BER_{k,n}\times {\text{ln}}^{2}\left(5BER_{k,n}\right)}\right)$$ (22)

As the gradient (∂*α*_*k*,*n*_/∂*BER*_*k*,*n*_) is always positive for the feasible BER range is 0 < *BER*_*k*,*n*_ < 0.02, it is clear that *α*_*k*,*n*_ is an increasing function of *BER*_*k*,*n*_.

## Supporting Information

S1 Dataset Comparison of different methods in terms of video quality. (XLSX)
