# Correction: Two-Level Scheduling for Video Transmission over Downlink OFDMA Networks

**DOI:** 10.1371/journal.pone.0152844

**Published:** 2016-05-18

**Authors:** Mau-Luen Tham, Chee-Onn Chow, Yi-Han Xu, Nordin Ramli

There are errors in the Funding section. The correct funding information is as follows: This project is supported by Introduction of high-level talents and overseas returnees scientific fund in Nanjing Forestry University (No. GXL015), and MIMOS Berhad. The funder MIMOS Berhad provided support in the form of salaries for author NR, but did not have any additional role in the study design, data collection and analysis, decision to publish, or preparation of the manuscript. The specific roles of these authors are articulated in the ‘author contributions’ section.

There are errors in the author affiliations. The affiliations should appear as shown here:

Mau-Luen Tham^4^, Chee-Onn Chow^1^, Yi-Han Xu^3^, Nordin Ramli^2^

1 Department of Electrical Engineering, Faculty of Engineering, University of Malaya, Kuala Lumpur, Malaysia, 2 Wireless Innovation, MIMOS Berhad, Technology Park Malaysia, Kuala Lumpur, Malaysia, 3 College of Information Science and Technology, Nanjing Forestry University, Nanjing, China, 4 Department of Electrical and Electronic Engineering, Lee Kong Chian Faculty of Engineering and Science, Universiti Tunku Abdul Rahman, Selangor, Malaysia
